# Ergonomic risk assessment in municipal waste management: integrating standardized methods with real-world context

**DOI:** 10.3389/fpubh.2026.1888499

**Published:** 2026-07-07

**Authors:** Vincenzo Cianci, Daniela Sapienza, Simone De Sio, Hector Nieto, Gabriella Martino, Concetto Mario Giorgianni

**Affiliations:** 1 Department of Biomedical and Dental Sciences and Morphofunctional Imaging, University of Messina, Messina, Italy; 2 Unit Research of Occupational Medicine, “Sapienza” University of Rome, Rome, Italy; 3 Occupational Medicine School, University of Business and Social Sciences (UCES), ABA, Buenos Aires, Argentina; 4 Department of Clinical and Experimental Medicine, University of Messina, Messina, Italy

**Keywords:** ergonomic risk assessment, manual material handling, NIOSH lifting equation, occupational health, Snook–Ciriello tables, sustainability, waste management, workplace safety

## Abstract

**Introduction:**

Manual handling of loads (MHL) represents a relevant occupational risk in the waste management sector, where lifting, pushing, and pulling are performed daily under variable and often complex operational conditions. This study aimed to assess ergonomic risk in a municipal waste management company through the application of standardized methods and to evaluate their utility for preventive decision-making in real-world contexts.

**Methods:**

This field-based ergonomic case study was conducted in a municipal waste management company. Ergonomic assessment worksheets were used to characterize two homogeneous door-to-door waste collection task scenarios: domestic collection and commercial collection. For lifting, worksheet-derived task duration, number of lifted objects, lifting frequency, load distribution, frequency/duration multipliers, and Lifting Index values were extracted and reported. Lifting indices were calculated according to the ISO 11228-1/ISO/TR 12295 framework using population-specific reference masses by sex and age group. Transport and towing components were assessed through worksheet-based indices derived from measured and recommended forces.

**Results:**

In the domestic collection scenario, two workers were involved: manual lifting lasted 180 min, with 182.5 objects lifted per worker and a lifting frequency of 1.014 lifts/min. Load distribution was mainly concentrated in the 5.5 kg category (82.2%), followed by 9.5 kg (16.4%) and 13.5 kg (1.4%). Lifting indices ranged from 0.746 in men aged 18–45 years to 1.243 in women aged >45 years. In the commercial collection scenario, one worker was involved: manual lifting lasted 120 min, with 60 objects lifted per worker, all in the 9.5 kg category, and a lifting frequency of 0.5 lifts/min. Lifting indices ranged from 0.442 in men aged 18–45 years to 0.737 in women aged >45 years. Towing indices remained below unity in both scenarios, with higher values for women during the maintenance phase.

**Discussion:**

Although most ergonomic indices remained within acceptable limits, the domestic collection scenario showed a subgroup-specific exceedance for women aged >45 years.

**Conclusion:**

Standardized ergonomic tools are useful for preventive planning in municipal waste management, provided that their results are interpreted in relation to actual task duration, load distribution, worker fitness for the task, and environmental variability. The findings support targeted preventive measures.

## 1 Introduction

Regulations associated with the “Circular Economy Package” have established progressive targets for preparing for the reuse and recycling of municipal waste (55% by 2025, 60% by 2030, and 65% by 2035) and a decrease in landfill disposal, making municipal waste management a strategic area for European environmental and industrial policies (1, 2). In this context, municipal waste management is increasingly influenced by sustainability policies and ongoing transformations in work organization, requiring adaptive approaches to occupational risk assessment in evolving operational environments (3–5).

However, according to Eurostat data, the problem is still quantitatively significant: in 2023, the EU’s municipal waste generation was 511 kg per person, whereas in 2022, the total amount of waste generated (including from households and all economic activities) exceeded 2,233 million tonnes (6, 7).

In this situation, municipal businesses and urban waste services are crucial to maintaining operator safety, operational continuity, and the quality of separate waste collection, often under real-world circumstances marked by unpredictability, time constraints, and interaction with intricate public areas (8). The waste industry poses a well-documented health and prevention challenge in addition to environmental concerns: lifting, carrying, pushing, and pulling activities are often inevitable and can result in biomechanical overload disorders (particularly musculoskeletal disorders of the spine and upper limbs) if performed in unfavorable ergonomic conditions (9–12). The problem’s centrality is in line with the overall epidemiological picture: low back pain is one of the main causes of disability globally; according to recent estimates, 619 million people will be affected in 2020, and projections show that number will rise by 2050 (8, 9, 13, 14). A sizable portion of the burden is also attributable to occupational factors. The World Health Organization (WHO) also highlights how low back pain affects social costs and work productivity (8). Evidence suggests that musculoskeletal disorders are still among the most prevalent work-related health issues in Europe. According to the European Agency for Safety and Health at Work (EU-OSHA) and European Working Conditions Survey (EWCS) summaries, musculoskeletal disorders remain among the most prevalent work-related health problems in Europe, with frequent involvement of the upper limbs and spine (4, 7, 15).

When narrowing the focus to waste collection and handling work, the scientific literature describes a high risk of musculoskeletal disorders and injuries, related to the combination of physical loads, awkward postures, and variable environmental conditions (4, 15–20). Studies of waste collection workers report significant prevalence rates of musculoskeletal disorders/pains: for example, a study of municipal waste collectors found a high proportion of workers with pain in at least one body region in the past year, with frequent involvement of the knee, shoulder, and lumbar spine (16). Other studies show overall rates of musculoskeletal complaints exceeding 50–60%, confirming that waste collection and handling is among the occupations with high biomechanical demands (16, 17, 21–24). Even in terms of accidents, international literature documents high rates of annual injury events in some cohorts of workers, highlighting how prevention must integrate ergonomic, organizational, and “traditional” safety aspects (cuts/punctures, slips, collisions, etc.) (21, 25–30).

A systematic review of the waste and recycling industry in Europe revealed higher rates of work-related illnesses and accidents (both fatal and non-fatal) in places like the UK than in other industries (25).

Legislative Decree 81/2008, which defines manual handling of loads as the collection of actions (lifting, putting down, pushing, pulling, carrying, transporting) that, because of the characteristics of the load or unfavorable ergonomic conditions, pose a risk of biomechanical overload injuries, is the primary source of information in Italian regulations (31).

Within the same regulatory framework, Article 168 emphasizes the use of technical standards and guidance tools; consistently, the study cites standards UNI ISO 11228-1 (lifting and transport), UNI ISO 11228-2 (pushing and pulling), UNI ISO 11228-3 (low loads at high frequency), and Technical Report ISO TR 12295 as an application guide for risk assessment and management. The guidelines of the National Prevention Plan also fit into this framework, proposing a phased process (identification, assessment, interventions, and reassessment) useful for structuring manual handling of loads (MHL) risk management over time (32, 33).

The study, therefore, aligns with the operational needs of waste management and the need for prevention based on validated tools. The sector, as discussed in the study, exposes workers to physically demanding tasks daily: handling heavy and bulky containers, transporting full wheeled trailers, working in confined spaces and in uneven terrain or in adverse weather conditions, factors that increase the likelihood of back pain, tendonitis, and joint injuries. Precisely this “field” variability makes it crucial to combine regulatory compliance with an ergonomic assessment capable of translating real-world conditions (grip geometry, distances, asymmetries, frequency, applied force) into quantitative indicators useful for defining priorities and corrective measures.

However, although standardized ergonomic assessment tools provide a structured and widely accepted approach for evaluating manual handling tasks, they are primarily based on simplified and relatively controlled task conditions. In complex occupational settings such as municipal waste management, characterized by high variability, dynamic task execution, and interaction with unpredictable environmental and organisational factors, these models may only partially capture the actual biomechanical exposure experienced by workers.

In this perspective, there is increasing recognition that task-based ergonomic indices should be interpreted within a broader framework that considers not only the mechanical characteristics of individual actions, but also the systemic interaction between worker-related factors, environmental constraints, and organizational dynamics. This highlights a potential gap between standardized risk assessment outputs and real-world exposure, suggesting the need for more integrative approaches in occupational health research and practice.

In this context, our study uses a municipal waste management company as a case study and focuses on the practical application of two widely recognized tools: the NIOSH method for lifting activities and the Snook and Ciriello tables for pushing and pulling (34, 35). The focus is on typical, concrete activities: in door-to-door waste collection, lifting and placing bags (with possible rotation of the trunk and a less-than-optimal grip), and in sweeping/collection activities, the movement of trolleys by pushing or pulling, often affected by slopes, differences in level, and route characteristics.

Beyond the application of standardized assessment methods, the study also aims to contribute to a more comprehensive interpretation of ergonomic risk in real-world settings, emphasizing how measured indices should be contextualized within the broader variability of operational conditions.

The overall objective, consistent with the study’s approach, is to demonstrate how the integrated use of standardized methods can support preventive decisions (targeted training, route maintenance, time management, aids, and best practices) and contribute to protecting the health of workers, in a sector destined to remain central in terms of the quantity of waste managed and the evolution of collection models.

## 2 Materials and methods

The assessment was designed as a field-based ergonomic case study of manual handling of loads in a municipal waste management company. The analysis focused on door-to-door waste collection activities and was based on ergonomic assessment worksheets completed during routine operating conditions (Table 1).

**Table 1:** Worksheet-derived task characteristics for the ergonomic assessment.

Scenario	Workers	Lifting duration	Lifted objects per worker	Lifting frequency	FM
Domestic collection	2	180 min	182.5	1.014 lifts/min	0.8796
Commercial collection	1	120 min	60	0.500 lifts/min	0.9700

FM = frequency/duration multiplier.

Two homogeneous task scenarios were considered in the analysis: (i) door-to-door domestic waste collection and (ii) door-to-door commercial waste collection. The domestic scenario involved two workers, whereas the commercial scenario involved one worker. Workers included in the assessment were active workers assigned to the evaluated tasks and judged fully fit for the job by the occupational physician. Workers with medical restrictions or limitations relevant to manual material handling were not assigned to these tasks and were therefore not included in the field assessment.

Musculoskeletal symptom questionnaires, injury records, absenteeism data, and follow-up data were not available. Therefore, the study was not designed to evaluate associations between ergonomic exposure and health outcomes. The sex- and age-specific categories used in the lifting analysis should be interpreted as population-based reference strata required by ISO/TR 12295, rather than as a description of the actual sex and age distribution of the observed workers.

For each homogeneous task scenario, worksheet-derived parameters were extracted, including number of workers involved, net duration of manual lifting, number of objects lifted per worker, lifting frequency, frequency/duration multiplier, load categories, percentage distribution of lifted objects, and lifting indices by ISO/TR 12295 sex- and age-specific reference groups. This approach was adopted to improve transparency and to address the variability of real field exposure more directly than a single simplified mono-task scenario.

Because the unit of analysis was the task scenario and not a sufficiently powered sample of individual workers, no inferential statistical analysis was performed. Results are therefore presented descriptively as deterministic ergonomic indices and interpreted for preventive planning and risk management.

### 2.1 Lifting assessment

For door-to-door collection, lifting was assessed using worksheet-derived exposure parameters. The analysis was based on the ergonomic assessment worksheets and included two homogeneous task scenarios: domestic door-to-door collection and commercial door-to-door collection (Table 2).

**Table 2:** Load distribution and ISO/TR 12295 Lifting Index values by sex- and age-specific reference group.

Scenario	Load distribution	Men 18–45	Women 18–45	Men >45	Women >45
Domestic collection	5.5 kg: 82.2%; 9.5 kg: 16.4%; 13.5 kg: 1.4%	0.746	0.932	0.932	1.243
Commercial collection	9.5 kg: 100%	0.442	0.553	0.553	0.737

Values >1 indicate conditions requiring preventive attention under the assessed task assumptions.

In the domestic collection scenario, the worksheet reported 180 min of manual lifting, 182.5 lifted objects per worker, and a lifting frequency of 1.014 lifts/min. The frequency/duration multiplier was 0.8796. The load distribution consisted of three categories: 5.5 kg, accounting for 82.2% of lifted objects; 9.5 kg, accounting for 16.4%; and 13.5 kg, accounting for 1.4%.

In the commercial collection scenario, the worksheet reported 120 min of manual lifting, 60 lifted objects per worker, and a lifting frequency of 0.5 lifts/min. The frequency/duration multiplier was 0.97. All lifted objects belonged to the 9.5 kg category.

Lifting risk was assessed according to the ISO 11228-1 / ISO/TR 12295 framework using RNLE-type multipliers and population-specific reference masses. The final Lifting Index values were reported for four ISO/TR 12295 reference groups: men aged 18–45 years, women aged 18–45 years, men aged >45 years, and women aged >45 years. These groups were used as standardized ergonomic reference categories for preventive interpretation and should not be interpreted as clinical or epidemiological subgroups of the observed workers.

The use of the original worksheet data allowed the analysis to account for actual task duration, lifting frequency, number of lifted objects, load distribution, and frequency/duration multipliers. This approach was adopted to reduce the potential underestimation of exposure associated with single low-frequency mono-task assumption and to provide a more transparent basis for ergonomic risk characterization and preventive planning (Table 3).

**Table 3:** Transport and towing indices extracted from the worksheets.

Scenario	Transport index 8 h	Transport index 1 h	Transport index 1 min	Towing initial men	Towing initial women	Towing maintenance men	Towing maintenance women
Domestic collection	0.138	0.000	0.367	0.340	0.437	0.367	0.633
Commercial collection	0.095	0.000	0.380	0.340	0.437	0.367	0.633

Transport indices are reported over 8 h, 1 h, and 1 min. Towing indices are reported separately for initial and maintenance phases and by sex-specific reference values. The sex- and age-specific categories are ISO/TR 12295 reference groups for ergonomic interpretation and not epidemiological subgroups of the observed workers.

Because the assessment was based on deterministic ergonomic models and task-level worksheet parameters, the resulting Lifting Index values were interpreted as ergonomic design and management indicators rather than as predictors of individual clinical outcomes.

### 2.2 Pushing/pulling assessment

For pushing and pulling bins/trolleys, the assessment was conducted using the Snook and Ciriello psychophysical tables (reference method for UNI ISO 11228-2), which allow comparison of the required effort with recommended values based on sex, distance of movement, frequency, and height of force application. Recommended forces were selected using the 75% “percent capable” criterion (i.e., the force acceptable to 75% of the relevant working population), matched to the observed task conditions. This approach enables the comparison of measured forces with population-based acceptability thresholds under standardized conditions, facilitating the identification of potentially critical task configurations.

For towing tasks, initial and maintenance forces were considered separately. Measured initial towing force was 5.5 kgf and measured maintenance force was 4.18 kgf. Recommended initial forces were 27 kgf for men and 21 kgf for women, whereas recommended maintenance forces were 19 kgf for men and 11 kgf for women. Accordingly, towing indices were calculated separately for initial and maintenance phases and by sex-specific reference values.

This reporting avoids relying on a single overall representative force and provides a more transparent interpretation of the task phases most likely to generate higher short-term mechanical demands. Nevertheless, the authors acknowledge that field measurements may not fully capture occasional peaks caused by slopes, curb transitions, uneven surfaces, obstacles, or abrupt maneuvers. This issue is explicitly discussed as a limitation.

Forces were measured with a dynamometer attached to the container/trolley, applying traction or push along a trajectory as horizontal as possible. To facilitate comparison with the Snook–Ciriello tables, readings were expressed in kilograms-force (kgf), with conversion from Newtons performed when required using kgf = N/9.81. In the analysis, initial and maintenance towing phases were reported separately rather than collapsed into a single overall representative value. The resulting towing indices were calculated as the ratio between measured force and recommended force for each sex-specific reference value and task phase. As with lifting indices, towing indices were interpreted as ergonomic planning indicators rather than as direct predictors of individual injury risk.

Finally, the analysis maintains a real-world perspective: the parameters used describe representative tasks, but it is recognized that environmental factors (uneven surfaces, slopes, obstacles, confined spaces, weather conditions, and load variability) can substantially modify actual effort. Therefore, the approach combines task observation, instrumental measurement of pushing/pulling, and standardized models (ISO 11228-1/ISO/TR 12295 for lifting; Snook–Ciriello tables per ISO 11228-2 for pushing/pulling) to obtain comparable indicators that can be used in prevention efforts (training, work organization, aids, route management, and targeted health surveillance), while recognizing that real-world variability may influence the actual biomechanical demands beyond those captured by standardized assessment conditions.

## 3 Results

### 3.1 Lifting exposure in door-to-door collection

Two homogeneous door-to-door collection scenarios were included in the worksheet-based analysis. The domestic collection scenario involved two workers and included 180 min of manual lifting during the shift. The number of lifted objects was 182.5 per worker, corresponding to a lifting frequency of 1.014 lifts/min. The frequency/duration multiplier was 0.8796. The distribution of lifted objects was mainly concentrated in the 5.5 kg category (82.2%), followed by the 9.5 kg category (16.4%) and the 13.5 kg category (1.4%).

In this domestic scenario, the ISO/TR 12295 lifting index was 0.746 for men aged 18–45 years, 0.932 for women aged 18–45 years, 0.932 for men aged >45 years, and 1.243 for women aged >45 years. Therefore, only the women aged >45 years reference group exceeded unity, indicating a condition requiring preventive attention under the assessed task assumptions.

The commercial collection scenario involved one worker and included 120 min of manual lifting during the shift. The number of lifted objects was 60 per worker, corresponding to a lifting frequency of 0.5 lifts/min. The frequency/duration multiplier was 0.97. All lifted objects belonged to the 9.5 kg category. In this scenario, the ISO/TR 12295 lifting index was 0.442 for men aged 18–45 years, 0.553 for women aged 18–45 years, 0.553 for men aged >45 years, and 0.737 for women aged >45 years. All values were below unity.

### 3.2 Transport and towing indices

Transport and towing indices were also extracted from the worksheets. In the domestic collection scenario, the cumulative transported mass index was 0.138 over 8 h, 0.000 over 1 h, and 0.367 over 1 min. In the commercial collection scenario, the corresponding values were 0.095 over 8 h, 0.000 over 1 h, and 0.380 over 1 min.

For towing, measured initial and maintenance forces were 5.5 kgf and 4.18 kgf, respectively. In men, the towing index was 0.340 for the initial phase and 0.367 for the maintenance phase. In women, the towing index was 0.437 for the initial phase and 0.633 for the maintenance phase. All towing indices were below unity, although maintenance towing in the female reference group showed the highest value and should therefore be considered more sensitive to worsening environmental conditions.

Overall, the worksheet-based results indicate that the domestic door-to-door collection scenario is the most critical condition, mainly because of longer lifting duration, higher lifting frequency, and a subgroup-specific exceedance among women aged >45 years.

## 4 Discussion

Overall, the worksheet-based analysis provides a more realistic description of a field exposure than a simplified mono-task scenario. The lifting exposure is represented by task-specific worksheet data including duration, number of objects lifted, frequency, load distribution, and frequency/duration multipliers.

The domestic collection scenario emerged as the most demanding condition. It involved 180 min of manual lifting, 182.5 lifted objects per worker, and a lifting frequency of approximately 1.014 lifts/min. Under these assumptions, the ISO/TR 12295 lifting index exceeded unity only in the women aged >45 years reference group (LI = 1.243). This finding should not be interpreted as a clinical diagnosis or as evidence of injury, but it should not be minimized either. It represents an ergonomic exceedance in a population-based reference group and therefore supports preventive attention, particularly through task redesign and exposure reduction (36–38).

The commercial collection scenario showed lower indices in all reference groups, consistent with shorter lifting duration, fewer lifted objects per worker, lower frequency, and a single 9.5 kg load category. Nevertheless, the comparison between the two scenarios illustrates that lifting risk in door-to-door waste collection is strongly influenced not only by the weight of individual objects, but also by the number of objects handled, task duration, frequency, and distribution of loads across the shift.

Towing indices remained below unity in both sex-specific reference groups. However, maintenance towing in the female reference group showed the highest index (0.633), suggesting greater sensitivity to unfavorable field conditions. Therefore, even when worksheet-derived indices are below recommended thresholds, route characteristics such as slopes, uneven surfaces, curb transitions, obstacles, wheel condition, friction, and maneuvering constraints may substantially increase the actual force required (39, 40).

These findings support a preventive interpretation of ergonomic indices. The results should be used to identify task configurations requiring attention, rather than to infer individual clinical risk or to conclude that the work is risk-free. This interpretation is particularly important in municipal waste collection, where exposure is dynamic, cumulative, and influenced by environmental and organizational variability (41, 42).

From a broader perspective, these findings support the need to interpret ergonomic risk within a more integrated, system-based framework. These aspects are particularly relevant in the context of ongoing transformations driven by sustainability goals and the progressive integration of technology in waste management systems.

While standardized methods such as the RNLE and Snook–Ciriello tables provide valuable quantitative indicators, they primarily capture task-specific mechanical demands under defined conditions. In real-world settings such as municipal waste collection, biomechanical exposure emerges from the dynamic interaction between multiple dimensions, including individual characteristics (e.g., age, sex, functional capacity), task-related factors (e.g., load, posture, frequency), environmental conditions (e.g., terrain, obstacles, urban layout), and organizational aspects (e.g., work pace, route design, temporal constraints).

Within this perspective, ergonomic risk should be considered not as a static property of isolated tasks, but as a context-dependent and cumulative phenomenon. This implies that even when individual task indices fall within acceptable limits, the overall exposure profile may still be relevant due to task variability, repetition, and combined physical demands over the work shift. Therefore, integrating task-based assessment with a system-level understanding of work organization and environmental variability may enhance the effectiveness of preventive strategies and support more comprehensive occupational health interventions.

In summary, these results provide a quantitative snapshot useful for prevention prioritization. The domestic door-to-door lifting task emerged as the most sensitive condition, particularly for the women aged >45 years ISO/TR 12295 reference group. Transport and towing indices remained below unity under the assessed worksheet-derived conditions, although maintenance towing in the female reference group showed the highest towing-related value and should therefore be interpreted cautiously in relation to route, surface, and equipment variability. The key message is that conclusions should remain prudent and contextual: standardized models are robust tools for planning, but municipal waste work is highly variable (32, 33, 43–47). Therefore, the most effective prevention strategy is not to assume that indices below unity correspond to negligible risk. Instead, these indicators should be used to guide targeted actions, including reducing lifting exposure, limiting excessive object accumulation, improving load organization, reducing high-frequency lifting sequences, using mechanical or organizational aids where feasible, optimizing routes, maintaining wheels and containers, reducing obstacles and slopes where possible, and providing task-specific training. These actions are particularly relevant for the domestic collection scenario, which showed the highest lifting indices and the only subgroup-specific exceedance.

### 4.1 Limitations and risk of bias

Several limitations should be acknowledged. First, this study is a field-based ergonomic case study conducted in one municipal waste management company and based on a small number of homogeneous task scenarios. Therefore, the findings should not be generalized to all waste collection systems, container types, vehicle designs, route configurations, or organizational models.

Second, the study was designed to assess ergonomic exposure and not health outcomes. No musculoskeletal symptom questionnaires, injury records, absenteeism data, or follow-up data were collected. Workers included in the assessment were active workers judged fully fit for the evaluated tasks by the occupational physician, while workers with medical restrictions relevant to manual material handling were not assigned to these tasks. Accordingly, the results cannot be used to estimate prevalence of musculoskeletal disorders or to test associations between ergonomic indices and clinical outcomes.

Third, individual demographic and occupational variables, such as exact age, sex distribution, and job experience, were not available for statistical analysis. The sex- and age-specific categories used in the lifting assessment are therefore ISO/TR 12295 reference groups for ergonomic interpretation, not epidemiological subgroups of the observed sample.

Fourth, the ergonomic indices are deterministic outputs derived from standardized assessment models and worksheet parameters. Inferential statistics, confidence intervals, and statistical hypothesis testing were not appropriate because the unit of analysis was the task scenario rather than a sufficiently powered sample of individual workers. To improve transparency, the manuscript reports task duration, lifting frequency, load distribution, and sex−/age-specific reference indices descriptively.

Finally, although the revised analysis uses worksheet-derived field data, real-world municipal waste collection remains highly variable. Load shape, grip quality, trunk rotation, confined spaces, slopes, surface irregularities, curb transitions, weather conditions, wheel maintenance, and time pressure may modify actual exposure. Therefore, the results should be interpreted as ergonomic planning indicators and integrated with contextual preventive assessment.

## 5 Conclusion

The worksheet-based ergonomic analysis indicates that door-to-door municipal waste collection presents different exposure profiles depending on task duration, number of objects lifted, lifting frequency, and load distribution. The domestic collection scenario was the most demanding condition, with a lifting index exceeding unity in the women aged >45 years ISO/TR 12295 reference group. The commercial collection scenario showed lifting indices below unity in all reference groups.

Transport and towing indices were below unity under the assessed conditions, although maintenance towing in the female reference group was the most sensitive towing condition and may worsen under unfavorable route or equipment conditions.

These findings support the usefulness of standardized ergonomic tools for preventive planning, but also highlight the need for contextual interpretation. Corrective actions should focus on reducing lifting exposure, limiting excessive object accumulation, improving load organization, maintaining wheels and containers, optimizing routes, reducing obstacles and slopes when possible, and providing task-specific training. Because no health outcome data were collected, conclusions are limited to ergonomic risk characterization and preventive prioritization, not to clinical or epidemiological inference.

## Data Availability

The original contributions presented in the study are included in the article/supplementary material, further inquiries can be directed to the corresponding author.

## References

[1] European Union. Directive (EU) 2018/851 of the European Parliament and of the Council of 30 May 2018 Amending Directive 2008/98/EC on Waste. Luxembourg: Off J Eur Union (2018). p. 109–40.

[2] EUR-Lex. EU waste management law (summary). (2025). Available online at: https://eur-lex.europa.eu/EN/legal-content/summary/eu-waste-management-law.html (Accessed December 11, 2025)

[3] Asibey MO Amponsah O Yeboah V. Solid waste management in informal urban neighbourhoods: occupational safety and health practices among tricycle operators in Kumasi, Ghana. Int J Environ Health Res. (2019) 29:702–17. doi: 10.1080/09603123.2019.1569211, 30714824

[4] Botti L Battini D Sgarbossa F Mora C. Door-to-door waste collection: analysis and recommendations for improving ergonomics in an Italian case study. Waste Manag. (2020) 109:149–60. doi: 10.1016/j.wasman.2020.04.027, 32408098

[5] Makundi H Simba CG Jahari C Tarmo C. Urban solid waste management and the decent work: insights from Tanzania's key cities. Waste Manag Res. (2025) 43:1663–75. doi: 10.1177/0734242X251329016, 40590406

[6] Eurostat. Waste statistics (statistics explained): total waste generated in the EU in 2022 = 2 233 million tonnes. (2024). Available online at: https://ec.europa.eu/eurostat/statistics-explained/index.php?title=Waste_statistics (Accessed December 15, 2025)

[7] Farioli A Mattioli S Quaglieri A Curti S Violante FS Coggon D. Musculoskeletal pain in Europe: the role of personal, occupational, and social risk factors. Scand J Work Environ Health. (2014) 40:36–46. doi: 10.5271/sjweh.3381, 24009006 PMC3964819

[8] World Health Organization Low back pain (fact sheet) (2023). Available online at: https://www.who.int/news-room/fact-sheets/detail/low-back-pain (Accessed December 20, 2025)

[9] GBD 2021 Low Back Pain Collaborators. Global, regional, and national burden of low back pain, 1990-2020, its attributable risk factors, and projections to 2050: a systematic analysis of the global burden of disease study 2021. Lancet Rheumatol. (2023) 5:e316–29. doi: 10.1016/S2665-9913(23)00098-X 37273833 PMC10234592

[10] Reddy EM Yasobant S. Musculoskeletal disorders among municipal solid waste workers in India: a cross-sectional risk assessment. J Family Med Prim Care. (2015) 4:519–24. doi: 10.4103/2249-4863.174270, 26985409 PMC4776602

[11] Yang CL Huang WP Lin WY Tseng PC Kuo HW. Job-related stress associated with work-related upper extremity musculoskeletal disorders (UEMDs) in municipal waste collectors: the moderation and mediation effect of job support. BMC Musculoskelet Disord. (2022) 23:762. doi: 10.1186/s12891-022-05721-y, 35948898 PMC9364533

[12] Ziaei M Choobineh A Abdoli-Eramaki M Ghaem H. Individual, physical, and organizational risk factors for musculoskeletal disorders among municipality solid waste collectors in shiraz, Iran. Ind Health. (2018) 56:308–19. doi: 10.2486/indhealth.2018-0011, 29503392 PMC6066438

[13] Graziosi F Bonfiglioli R Decataldo F Violante FS. Criteria for assessing exposure to biomechanical risk factors: a research-to-practice guide-part 1: general issues and manual material handling. Life (Basel, Switzerland). (2024) 14:1398. doi: 10.3390/life14111398, 39598195 PMC11595560

[14] Graziosi F Bonfiglioli R Decataldo F Violante FS. Criteria for assessing exposure to biomechanical risk factors: a research-to-practice guide-part 2: upper limbs. Life (Basel, Switzerland). (2025) 15:109. doi: 10.3390/life15010109, 39860049 PMC11767204

[15] Acquah AA D'Souza C Martin B Arko-Mensah J Quakyi IA Basu N . Work-related exposures and musculoskeletal disorder symptoms among informal E-waste recyclers at Agbogbloshie, Ghana. Proc Congr Int Ergon Assoc. (2021) 222:677–81. doi: 10.1007/978-3-030-74611-7_93, 34308445 PMC8301232

[16] European Agency for Safety and Health at Work (EU-OSHA). Musculoskeletal disorders (topic page). (2025). Available online at: https://osha.europa.eu/en/themes/musculoskeletal-disorders (Accessed December 22, 2025)

[17] GBD 2021 Diseases and Injuries Collaborators. Global incidence, prevalence, years lived with disability (YLDs), disability-adjusted life-years (DALYs), and healthy life expectancy (HALE) for 371 diseases and injuries in 204 countries and territories and 811 subnational locations, 1990-2021: a systematic analysis for the global burden of disease study 2021. Lancet. (2024) 403:2133–61. doi: 10.1016/S0140-6736(24)00757-8, 38642570 PMC11122111

[18] Degli Esposti A Magrini C Bonoli A. Door-to-door waste collection: a framework for the socio-economic evaluation and ergonomics optimisation. Waste Manag. (2023) 156:130–8. doi: 10.1016/j.wasman.2022.11.024, 36462343

[19] Löfqvist L Osvalder AL Bligård LO Pinzke S. An analytical ergonomic risk evaluation of body postures during daily cleaning tasks in horse stables. Work. (2015) 51:667–82. doi: 10.3233/WOR-152022, 26409939

[20] Salve PS Chokhandre P. Assessing the exposure of street sweeping and potential risk factors for developing musculoskeletal disorders and related disabilities: a cross-sectional study. BMJ Open. (2016) 6:e012354. doi: 10.1136/bmjopen-2016-012354, 27986735 PMC5168656

[21] Abou-Elwafa HS El-Bestar SF El-Gilany AH Awad Ee-S. Musculoskeletal disorders among municipal solid waste collectors in Mansoura, Egypt: a cross-sectional study. BMJ Open. (2012) 2:e001338. doi: 10.1136/bmjopen-2012-001338, 22977187 PMC3467652

[22] Idrees A Kashif M Kompal R Umar A Nadeem I Fatima R. Musculoskeletal discomfort and wrist flexor tendonitis among street sweepers and associated risk factors. Work. (2023) 76:1395–405. doi: 10.3233/WOR-220253, 37393466

[23] van Kampen V Hoffmeyer F Seifert C Brüning T Bünger J. Occupational health hazards of street cleaners - a literature review considering prevention practices at the workplace. Int J Occup Med Environ Health. (2020) 33:701–32. doi: 10.13075/ijomeh.1896.01576, 32939096

[24] Netto K Francis-Pester G Lewis C Dunnill P Darling R. Posture and muscle activity during waste collection work. Annals Work Exposures Health. (2022) 66:119–23. doi: 10.1093/annweh/wxab022, 34347044

[25] Poole CJM Basu S. Systematic review: occupational illness in the waste and recycling sector. Occupational Med. (2017) 67:626–36. doi: 10.1093/occmed/kqx153, 29165683 PMC5927023

[26] Ephraim P Stephens JK Myers-Hansen GA Otwey RY Amon S Kporxah MK . Prevalence and determinants of occupational injuries among solid waste collectors of Zoomlion Ghana limited. J Environ Public Health. (2021) 2021:1. doi: 10.1155/2021/6914529, 35003273 PMC8741403

[27] Sara HH Bayazid AR Quayyum Z. Occupational health sufferings of child waste Workers in South Asia: a scoping review. Int J Environ Res Public Health. (2022) 19:8628. doi: 10.3390/ijerph19148628, 35886477 PMC9316577

[28] Temesgen LM Mengistu DA Mulat S Mulatu G Tolera ST Berhanu A . Occupational injuries and associated factors among municipal solid waste collectors in Harar town, eastern Ethiopia: a cross sectional study. Environ Health Insights. (2022) 16:11786302221104025. doi: 10.1177/11786302221104025, 35719847 PMC9201305

[29] ZhuJ FletcherA VermaN. Fatal injuries associated with rear step riding among municipal solid waste collection workers (United States, 1984-2020). Am J Ind Med. (2023) 66:1069–78. doi: 10.1002/ajim.23537, 37746824

[30] Eskezia D Aderaw Z Ahmed KY Tadese F. Prevalence and associated factors of occupational injuries among municipal solid waste collectors in four zones of Amhara region, Northwest Ethiopia. BMC Public Health. (2016) 16:862. doi: 10.1186/s12889-016-3483-1 27554260 PMC4995673

[31] Repubblica Italiana. Decreto Legislativo 9 aprile 2008, n. 81 (Testo Unico salute e sicurezza sul lavoro) – Art. 168 (Titolo VI: Movimentazione manuale dei carichi). Rome: Normattiva. (2028). Accessed 2 Jan 2026

[32] ISO. ISO/TR 12295:2014 Ergonomics—Application Document for ISO Standards on Manual Handling (ISO 11228 series) and Working Postures (ISO 11226). Geneva: International Organization for Standardization (2014).

[33] ISO. ISO 11228-1:2021 Ergonomics—Manual Handling—Part 1: Lifting and Carrying. Geneva: International Organization for Standardization (2021).

[34] Waters TR Putz-Anderson V Garg A. Applications manual for the revised NIOSH lifting equation (updated manual) (2021). Available online at: https://stacks.cdc.gov/view/cdc/110725 (Accessed January 15, 2025)

[35] Snook SH Ciriello VM. The design of manual handling tasks: revised tables of maximum acceptable weights and forces. Ergonomics. (1991) 34:1197–213. doi: 10.1080/00140139108964855, 1743178

[36] Leone GD Cocchini A Graziano R Broccoli M Sulli D Sferrazzo F . Piano Nazionale della Prevenzione 2014–2018. Linee di indirizzo per l’applicazione del Titolo VI del D.Lgs. 81/08 e per la valutazione e gestione del rischio connesso alla Movimentazione Manuale di Carichi (MMC). Rome: Testo definitivo (2016).

[37] Fox RR Lu M-L Occhipinti E Jaeger M. Understanding outcome metrics of the revised NIOSH lifting equation. Appl Ergon. (2019) 81:102897. doi: 10.1016/j.apergo.2019.102897, 31422239

[38] Waters TR Putz-Anderson V Garg A Fine LJ. Revised NIOSH equation for the design and evaluation of manual lifting tasks. Ergonomics. (1993) 36:749–76. doi: 10.1080/00140139308967940, 8339717

[39] Asante BO Trask C Adebayo O Bath B. Prevalence and risk factors of low back disorders among waste collection workers: a systematic review. Work. (2019) 64:33–42. doi: 10.3233/WOR-192977, 31450537

[40] Engkvist IL Svensson R Eklund J. Reported occupational injuries at Swedish recycling centres - based on official statistics. Ergonomics. (2011) 54:357–66. doi: 10.1080/00140139.2011.556261, 21491278

[41] Tshivhase SE Mashau NS Ngobeni T Ramathuba DU. Occupational health and safety hazards among solid waste handlers at a selected municipality South Africa. Health SA. (2022) 27:1978. doi: 10.4102/hsag.v27i0.1978, 36570087 PMC9772716

[42] Jeong BY Lee S Lee JD. Workplace accidents and work-related illnesses of household waste collectors. Saf Health Work. (2016) 7:138–42. doi: 10.1016/j.shaw.2015.11.008, 27340601 PMC4909843

[43] ISO. ISO 11228-2:2007 Ergonomics — Manual Handling — Part 2: Pushing and Pulling. Geneva: International Organization for Standardization; (2007).

[44] Potvin JR Ciriello VM Snook SH Maynard WS Brogmus GE. The liberty mutual manual materials handling (LM-MMH) equations. Ergonomics. (2021) 64:955–70. doi: 10.1080/00140139.2021.1891297 33729096

[45] Jung MC Haight JM Freivalds A. Pushing and pulling carts and two-wheeled hand trucks. Int J Ind Ergon. (2005) 35:79–89. doi: 10.1016/j.ergon.2004.08.006

[46] Al-Eisawi KW Kerk CJ Congleton JJ Amendola AA Jenkins OC Gaines W. Factors affecting minimum push and pull forces of manual carts. Appl Ergon. (1999) 30:235–45. doi: 10.1016/S0003-6870(98)00019-2, 10327087

[47] Garg A Waters T Kapellusch J Karwowski W. Psychophysical basis for maximum pushing and pulling forces: a review and recommendations. Int J Ind Ergon. (2014) 44:281–91. doi: 10.1016/j.ergon.2012.09.005, 26664045 PMC4672999

